# Information about different treatment options and shared decision making in dialysis care - a retrospective survey among hemodialysis patients

**DOI:** 10.1186/s12913-021-06599-7

**Published:** 2021-07-08

**Authors:** Isabell Schellartz, Tim Ohnhaeuser, Thomas Mettang, Nadine Scholten

**Affiliations:** 1 Faculty of Human Sciences, Faculty of Medicine, Institute of Medical Sociology, University of Cologne, Health Services Research and Rehabilitation Science (IMVR), Eupener Str. 129, 50933 Cologne, Germany; 2 Kidney Center, Wiesbaden, Germany

**Keywords:** Information about different treatment options, Informed Choice, Hemodialysis, Chronic Kidney Disease, Shared Decision making

## Abstract

**Background:**

Hemodialysis (HD) and peritoneal dialysis (PD) are equivalent treatment alternatives for patients with end stage renal disease. In Germany, there is a legal obligation to inform every patient about all treatment alternatives and their possible harms and benefits. However, there is a low utilization of PD. Therefore, the question arises, whether HD patients perceive that they were informed about different dialysis options. We further investigate, if personal characteristics of informed and non-informed patients vary, and if both groups experienced the decision for their dialysis treatment as shared decision making (SDM).

**Methods:**

The database was a nationwide postal survey of 590 HD patients from two statutory health insurers in Germany. Participants were asked whether they have been informed about both dialysis options. A logistic regression model examines impact factors on this information. We investigate differences in the German version of the 9-item SDM Questionnaire (SDM-Q-9) between informed and non-informed patients with a multivariate linear regression model.

**Results:**

56 % of the respondents reported they had been informed about different dialysis treatment options. Patients older than 65 had a 61 % lower chance than patients ≤ 65 for this information (*p* < 0.001). High educated patients had a 47 % higher chance for this information than patients with low education level (*p* = 0.030). Informed patients rated a higher SDM-Q-9 scores than non-informed patients (76.9 vs. 44.2; *p* < 0.001). Non-informed patients showed high values in those SDM-Q-9 items which had no regard to different treatment options.

**Conclusions:**

A great proportion of HD patients – mostly elderly patients and patients with a low education level – did not perceive that they were informed about different dialysis options before dialysis was initiated. The current obligation to provide information about all treatment alternatives in Germany is a first step to assure the unselected access to different treatment options. But it has not reached routine application in health care yet. Information about different treatment options can pave the way for SDM. While SDM is considered to be a valuable tool in clinical medicine, there is still room for improvement for its successful implementation when it comes to decision making on different dialysis treatment options.

**Trial registration:**

The MAU-PD study (Multidimensional analysis of causes for the low prevalence of ambulatory peritoneal dialysis in Germany) is registered at the German Clinical Trials Register.

- DRKS-ID: DRKS00012555
- Link: https://www.drks.de/drks_web/setLocale_EN.do.
- Date of Registration in DRKS: 2018/01/04.

## Background

### Shared decision making and information about different treatment options

Shared decision making (SDM) aims to ensure patients are informed about their medical conditions, treatment options and resulting benefits and harms, so that they can participate in their medical decision making [1]. A shared decision requires both parties to be informed about the possible aspects relevant for the treatment decision [2]. This also includes information about all treatment alternatives. Thus, in Germany, there is a legal obligation to inform every patient about all treatment alternatives and their possible harms and benefits. This information also has to be given in a way that the patient can understand [3]. The policy aims to enable an unselected access to healthcare. Patients can weigh the advantages and disadvantages according to their personal preferences, values and goals. Therefore, SDM is seen as fundamental to patient empowerment and patient-centered care [4]. Kayyali et al. reported, that treatment information and SDM is not always provided optimally to patients with chronic conditions [5]. SDM is particularly relevant for patients suffering from life-changing chronic diseases, where two or more equivalent treatment options can be offered [6].

### Choice of dialysis treatment

Patients with end stage renal disease (ESRD) are faced with deciding between different treatment options when they reach a certain point in the progression of their disease [7]. They suffer from a series of clinical problems, including intoxication by uremic toxins. As a symptomatic treatment, dialysis can be installed to sustain the patient’s life. There are currently two different dialysis options: hemodialysis (HD) and peritoneal dialysis (PD). With the HD treatment, the patient’s blood is filtered with a machine, usually three times per week for four hours in an ambulatory dialysis center. In PD, the peritoneal membrane is used as an ‘inner filter’ which allows permeation of toxins and water into a dialysis solution within the peritoneal cavity. This solution is administered via a peritoneal catheter and has to be replaced by the patient. This can be done manually four times a day; at home, at work, or in any suitable location. It is also possible to connect a cycler at night that replaces the dialysis solution. Dialysis is performed during the sleep then and patients have a ‘free’ day. Most patients are eligible for both dialysis options [8, 9]. HD and PD are medically equivalent in terms of survival [10–12]. The provision of information about different dialysis treatment options varies among dialysis centers. They can use decision aids or training classes. It may be possible to meet an experienced patient.

In Germany, there is a low PD ratio of 7 % [13]. Robinski and colleagues published that in Germany PD patients report higher SDM scores than HD patients [14]. Investigations also showed that predialysis patient education, decision aids and SDM interventions can increase the PD uptake [15, 16]. They also improve the decision making process for a renal replacement therapy [17–19]. Hence, the relevance of SDM-differences between HD and PD patients has been demonstrated. This article takes a closer look at HD patients’ perception of the information they received about different treatment options and the decision making process. Have HD patients been informed in the dialysis center about different dialysis treatment options? Do personal characteristics of informed and non-informed patients vary? And were informed patients more likely to experience their decision process for their dialysis treatment as SDM?

## Methods

### Study design and setting

A retrospective cross-sectional study among dialysis patients was conducted. So, we investigated the real-world setting of the decision making process for the dialysis treatment. This examination was part of the MAU-PD study (Multidimensional analysis of causes for the low prevalence of ambulatory peritoneal dialysis in Germany). It aimed to find possible reasons for the low PD proportion in Germany from patients’, physicians’ and nurses’ perspectives [20].

### Data collection and study population

The majority of the German population (90 %) are members of a statutory health insurance (SHI) [21]. SHIs are obliged to contract every person and thus have a broad collective of insurants [22]. A collaboration with two large SHIs allowed a survey among their insurants. DAK-Gesundheit (Deutsche Angestellten-Krankenkasse) and SBK (Siemens Betriebskrankenkasse) together cover 6.6 million insurants [23, 24]. They contacted their adult insurants on dialysis for a nationwide postal survey at the end of 2018/beginning of 2019. The questionnaire, study information, and a franked envelope for this postal survey had been prepared by the study group before. Participants returned the completed questionnaire to the study group anonymously. Having an anonymous survey design, participants gave their informed consent to participate and publish the summarized results by returning the completed questionnaire. They were informed about this procedure in the written study information. We reminded once.

There is a possibility of a recall bias about the situation prior to the first dialysis with patients having a large time since their initial dialysis (dialysis vintage). Therefore, respondents with a dialysis vintage longer than three years were excluded. Due to the selection via two SHIs, the study population consists of patients from different dialysis centers, who provide the information about different dialysis treatment options differently.

### Measures

The German version of the validated and widely used 9-item Shared Decision Making Questionnaire (SDM-Q-9) was applied [25]. It measures patients’ retrospective perception of SDM in clinical encounters [25]. Different languages of it have been used in the context of ESRD before [14, 26]. The nine items of the SDM-Q-9 consider different aspects of the decision making process on a 6-point Likert scale from 0 “completely disagree” to 5 “completely agree” [25]. Hence, single item values from 0 to 2 mean a tendency to disagree, values higher than 2 mean a tendency to agree. Due to the positive wording, high values correspond to a high degree of SDM. A Cronbach’s alpha of 0.938 in the validation study emphasizes the internal consistency of the measure [25]. In order to refer to the dialysis care, we changed the items’ wording slightly from “my physician” to “my dialysis physician”. In the introductory phrase, we also pointed out that the items should refer to discussions with their dialysis physician about their upcoming dialysis. The SDM-Q-9 does not contain an explicit question about the information patients received about different treatment options. Therefore, we developed an additional single-item: “Were you informed in the dialysis center that there are two fundamentally different dialysis options (hemodialysis and peritoneal dialysis)?”. Answer categories were “yes” and “no.” Information on the respondents’ age, sex and education level as well as the dialysis vintage was collected. The education level was measured by the school education level with answer categories ‘no school education’ or ‘basic school qualification’, ‘extended secondary school diploma’ or ‘A levels’.

### Data analysis

Education level was binary coded in ‘low’ (no or basic school qualification) or ‘high’ (extended secondary school diploma or A levels) education level. This makes the German levels internationally understandable and comparable. Age was binary coded in > 65 years and ≤ 65 years. Descriptive results present the percentage of respondents, who stated they had been informed about different dialysis treatment options. Single chi square tests investigate, whether informed and non-informed participants vary in sex, age and education level. The difference in the dialysis vintage between informed and non-informed participants is examined by a Wilcoxon-Man Whitney test. A multivariate logistic regression model with the dependent variable information about different dialysis treatment options (yes/no) was built. Odds ratios for the chance for information about different dialysis treatment options were calculated for the independent variables age, sex and education level.

As instructed by the authors, composite scores of the SDM-Q-9 scale are presented on a 0-100 scale; higher composite scores represent a higher degree of SDM [25]. The aim was to investigate SDM-Q-9 differences between informed and non-informed patients. So, it was important to precisely display all different aspects of SDM with respect to all possible types of bias. Hence, we only calculated composite scores for participants who responded to all nine items. A Wilcoxon Mann Whitney test compared mean SDM scores of informed and non-informed patients. In a multivariate linear regression model, this effect is adjusted for age and education level. Statistical computations were conducted in Stata 16.

## Results

### Descriptive sample characteristics

**Fig. 1:**
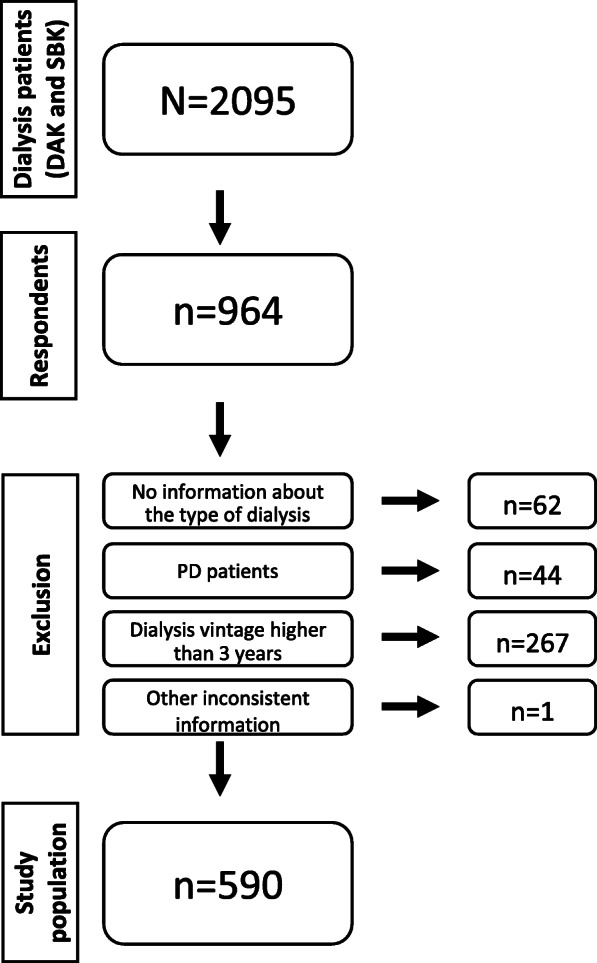
Data collection flowchart

A total of 964 dialysis patients responded to the questionnaire. This means a response rate of 46 %. Figure 1 provides an overview about exclusion criteria and our study population. The characteristics of 590 included HD patients are illustrated in Table 1. 60 % were female and 72 % older than 65 years. 51 % had a high education level. The mean dialysis vintage was 2 years.

**Table 1:** Patient characteristics

		Total n [%]	Informed n [%]	Non-informed n [%]
**Sex**				
	Male	353 [60.1]	204 [58.8]	143 [41.2]
	Female	234 [39.9]	119 [51.7]	111 [48.3]
**Age**				
	≤ 65 years	168 [28.5]	121 [72.5]*	46 [27.4]*
	> 65 years	422 [71.5]	203 [49.2]*	210 [50.8]*
**Education level**				
	low	285 [49.2]	137 [49.3]**	141 [50.7]**
	high	294 [50.8]	182 [62.3]**	110 [37.7]**
**Dialysis vintage in years, mean [SD]**		2.0 [0.5]	2.0 [0.6]	2.0 [0.5]

Notes: Standard deviation (SD); *p < 0.001, **p = 0.002 (single chi square tests)

### Information about different dialysis treatment options

56 % of HD patients reported they received information about different dialysis treatment options. 49 % of participants older than 65 stated they received this information (vs. 72 % ≤65 years, *p* < 0.001). 49 % of those with low education level perceived they were informed, as were 62 % of those with high education level (*p* = 0.002). The multivariate logistic regression model displayed in Table 2 shows age and education level had a significant impact on the chance for information about the different dialysis treatment options. Participants older than 65 had a 61 % lower chance to receive this information (*p* < 0.001; 95 % CI 0.26–0.58). Patients with a high education level had a 47 % higher chance for information about different dialysis treatment options compared to participants with a low education level (*p* = 0.030; 95 % CI 1.04–2.07). Pseudo R² was 0.044.

**Table 2:** Results from the multivariate logistic regression model on information (no/yes)

		Odds ratio	*p*-value	95 % Confidence interval
**Constant**		**1.77**	**0.010**	**1.15–2.72**
**Age**				
	> 65 years Reference: ≤ 65 years	**0.39**	**< 0.001**	**0.26–0.58**
**Education level**				
	high Reference: low	**1.47**	**0.030**	**1.04–2.07**
**Sex**				
	Male Reference: female	1.32	0.126	0.93–1.87

### SDM in the decision for the dialysis treatment

**Fig. 2:**
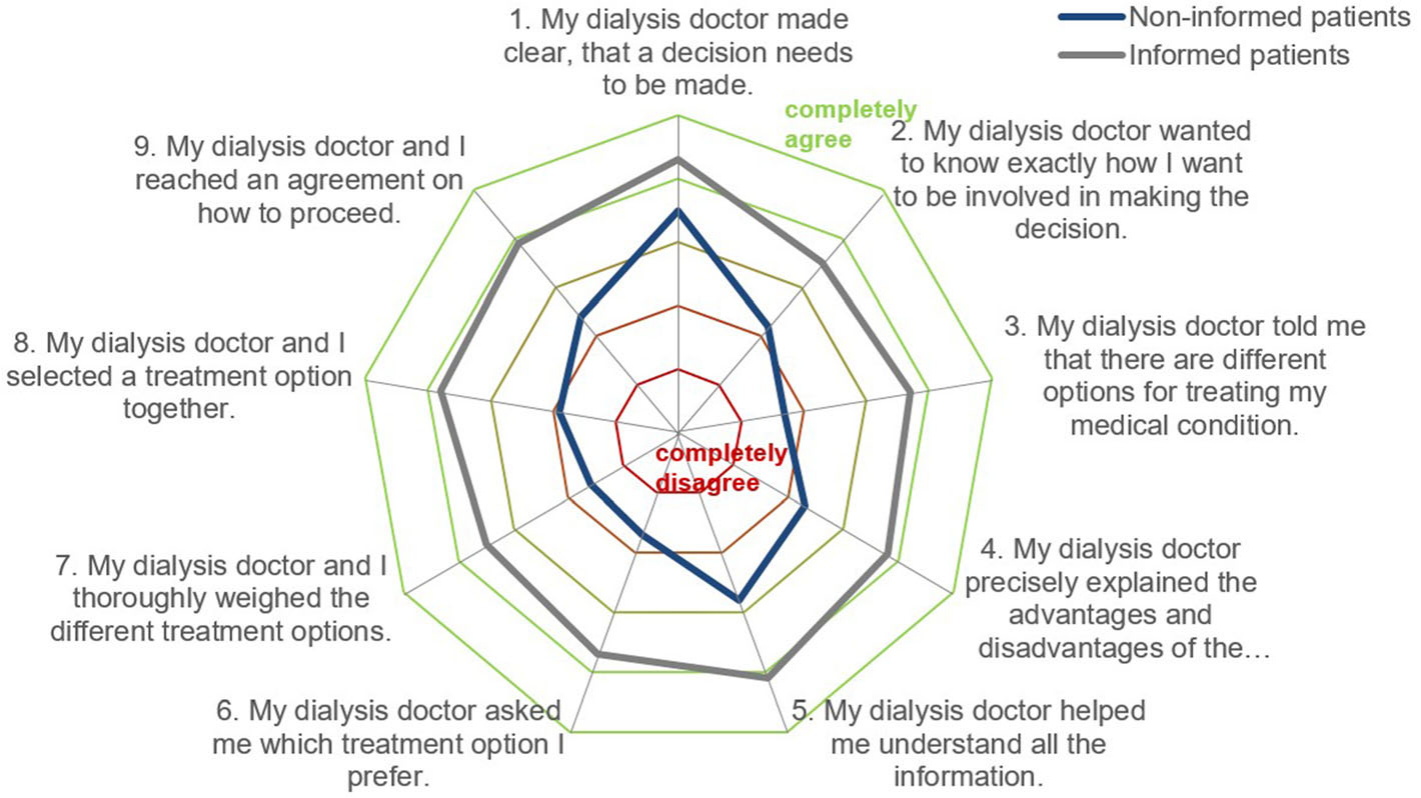
Mean SDM item values of informed and non-informed patients. Notes: The different items are presented on axes starting from a shared point in the middle. This middle point represents the lowest answer category “completely disagree”, while the outer line stands for the highest answer category “completely agree”. Mean item values of informed and non-informed patients are displayed on each item’s axis

The radar chart in Fig. 2 illustrates all nine SDM-Q-9 items and differences in its response behavior between informed and non-informed patients (information about different dialysis treatment options). A tendency to agree with the item showed non-informed patients with the items no. 1 and 5. Besides item no. 2, these are also the SDM items with the least difference between informed and non-informed patients. The biggest difference is between items no 3 and 6. Informed patients rated those items on average 2 points higher on the 6-point Likert scale. All SDM-Q-9 items show significant differences between informed and non-informed patients (*p* < 0.001).

**Fig. 3:**
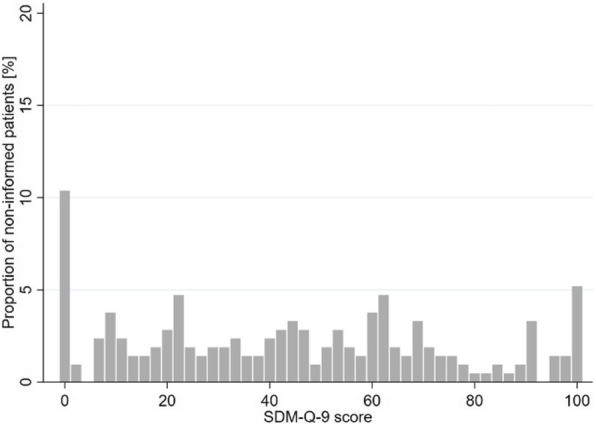
Distribution of SDM-Q-9 scores of non-informed patients

**Fig. 4:**
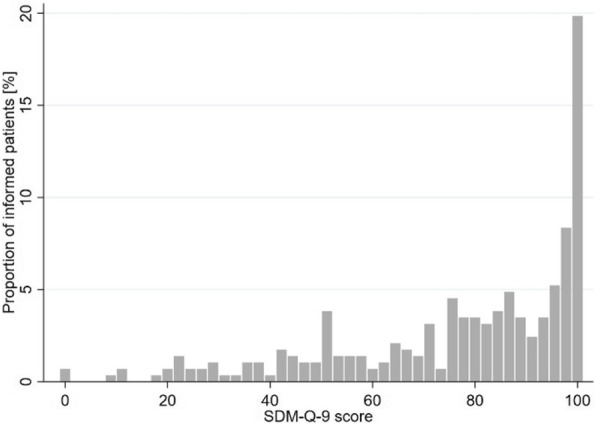
Distribution of SDM-Q-9 scores of informed patients

Cronbach’s alpha of the SDM-Q-9 was 0.94 in our study. Figures 3 and 4 show the distribution of composite SDM-Q-9 scores of informed and non-informed patients. Non-informed patients’ composite scores on SDM-Q-9 varied, whereas a high proportion of informed patients experienced SDM (left-skewed distribution). Comparing the mean composite SDM-Q-9 scores of both groups, informed patients rated higher SDM-Q-9 scores than the non-informed patients (76.9 vs. 44.2; *p* < 0.001; Wilcoxon Mann-Whitney test). This effect remains significant when adjusted for age and education level in a multivariate linear regression model (Table 3). In this model, informed patients exhibited 33 points higher SDM-Q-9 scores (95 % CI: 28.20–38.20; *p* < 0.001). The confounders age and education level showed no significant effect on SDM-Q-9 scores. R² is 0.270.

**Table 3:** Results from the multivariate linear regression model on the SDM-Q-9

		Coefficient	*p*-value	95 % Confidence interval
**Constant**		**41.27**	**< 0.001**	**35.08–47.5**
**Information**				
	Informed Reference: non-informed	**33.29**	**< 0.001**	**28.4–38.02**
**Age**				
	> 65 years Reference: ≤ 65 years	3.45	0.199	-1.82–8.73
**Education level**				
	high Reference: low	0.41	0.869	-4.43–5.25

## Discussion

This article aimed to investigate, whether all HD patients have been informed about different dialysis treatment options, if there are factors influencing the chance for information and if they experienced their decision process for their dialysis treatment as SDM.

A high proportion of HD patients (44 %) did not feel they have received information about different dialysis treatment options. Younger and higher educated patients had a higher chance to perceive they have been informed. Informed patients rated higher SDM-Q-9 scores treatment decision than non-informed patients. Non-informed patients showed high values in those SDM-Q-9 items which had no regard to different treatment opportunities.

Participants younger than 65 had a 61 % higher chance for perceiving they had been informed about different dialysis treatment options. These results correspond with those of Machowska et al. They also reported patients older than 69 had a 60 % lower chance to receive structured education about dialysis options [27]. High age is not a contraindication against PD [8, 9]. But with increasing age the probability of having a comorbidity which is relevant for the decision on a certain dialysis option rises. Therefore, it could be possible that during the decision making process it becomes clear that a medical contraindication to either PD or HD exists. This can lead the treating nephrologist to refrain from informing the patient about a treatment option that is obviously not suitable. Although there are only a few medical contraindications to PD [8, 9], higher age with compromised capabilities for daily work and reduced cognitive and physical performance is felt to be a contraindication to PD treatment. Therefore, a nephrologist may consider an older patient as unsuitable, although age is not an absolute contraindication to PD. The higher proportion of missing information about different dialysis treatment options in older patients can help to explain why HD patients are often older than PD patients in several study populations [28–30].

The legal obligation to inform about all treatment alternatives and provide this information in a understandable way [3] tries to assure a non-biased access to healthcare. But it seemingly does not prevent from a certain selection of who receives information in a way that is perceived and remembered. Nephrologists are probably unaware of this selection. Not every nephrologist provides PD, which could make it difficult for them to offer information about this treatment opportunity. The provision of equal and independent information as intended by law is difficult to utilize. To be able to implement this in everyday health care, better framework conditions for professionals might be helpful: neutral and independent decision aids and sufficient time. This is needed, because the extent to which combinations of therapeutic benefits and side effects of treatment opportunities are perceived as advantages or disadvantages can vary greatly among dialysis patients [31].

Pseudo R² of the multivariate logistic regression reports a limited predictable variance of 4 %. Receiving information about different dialysis treatment options seemingly is not only dependent on the predictors in our multivariate model. There is sociodemographic selection is not the only reason for not receiving information. There can be medical contraindications, certain routines in one dialysis center to provide the information about different dialysis treatment options or other confounders. It is also possible that the patient is already uremic by the acute intoxication and needs urgent dialysis. This makes it difficult to provide information or may be a reason for patients not remembering they received information about different treatment options.

Our results show a positive association between being informed about different dialysis treatment options and reporting a higher degree of SDM. Patients who did not receive information about different dialysis treatment options had mean SDM scores of 44.2. This means a tendency to disagree with the items. Hence, non-informed patients on average did not perceive the decision that was made about their type of dialysis as a shared one. Considering the foundation for participating in the treatment process – the information about both dialysis treatment options – is not given in this group, the mean SDM score still seems quite high. As Figs. 3 and 4 illustrate, SDM scores vary greatly between informed and non-informed patients. While a large part of the informed patients gave very high SDM scores (left-skewed distribution), the SDM rating varied greatly among the non-informed patients. The different distributions confirm that equal information transfer is fundamental and highly important for SDM.

The differences between the single items confirm this assumption (see Fig. 2). Non-informed patients state relatively high values in items 1 and 5. These items do not refer to different treatment options but to the relevance of a decision and help with information, which can easily refer to the timing of the initial dialysis. These items can be responsible for the relatively high SDM-Q-9 scores, although there actually was no shared decision about treatment alternatives. Items 3 and 6 show the biggest difference. Indeed, these items refer to different dialysis treatment options.

The legal obligation to inform about all treatment alternatives and SDM target a non-biased access to different treatment options and a treatment decision with regards to patients’ preferences. In the context of dialysis care, it does not mean increasing the PD rate. Therefore, we investigated HD patients. Our results show, that there is still a number of HD patients, who did not know about other treatment alternatives and thus did not have the opportunity to choose a dialysis treatment with regard to their preferences.

### Strengths and limitations

Our study population consisted of 590 HD patients out of 70,400 HD patients insured by SHIs [13]. Selecting patients from a large and broad collective of SHI insurants and having a high response rate of 46 % are strengths of our study.

The participants stated ‘yes’ or ‘no’ regarding whether they had received information about different dialysis treatment options in the treating dialysis center. Patients have different dialysis vintages. Hence, there is a potential recall bias in remembering whether they received information about different dialysis treatment options or if it was a shared decision. In addition, the patients may have been uremic when they received this information. In order to mitigate this potential bias, we have excluded those patients who initiated dialysis more than three years ago. Another recall bias can occur with regard to the participant’s age. Due to potentially reduced cognitive function, older patients may not remember the information they received. This potential bias may contribute older patients to have a lower chance to report they were informed about different dialysis treatment options.

The SDM-Q-9 is a validated, widely used instrument [25] that also shows high reliability in our study (Cronbach’s alpha = 0.94). It refers to different aspects of decision making. Participants were asked to relate the SDM-Q-9 items to their upcoming dialysis. Our wording in the introductory phrase may have led participants to assume that the construct refers to the decision about the timing of the initial dialysis. But some of the SDM items explicitly address different treatment options, which cannot refer to the decision about the initial dialysis. Notwithstanding the above, there are no hints in our sample that the understanding of the SDM-Q-9 items varies between informed and non-informed patients. If this bias exists, it is probably uniformly distributed and does not bias our results. A closer look at the single items shows that the biggest difference between informed and non-informed patients occurs in those items which refer to different treatment options. This also confirms our assumption that the potential misunderstanding of our introductory phrase does not bias our results.

## Conclusions

A large proportion of HD patients – mostly elderly patients and patients with a low education level – stated that they did not receive information about different dialysis treatment options. Non-informed patients tended not to experience SDM in the decision process for their dialysis treatment. Both, the legal obligation to inform patients about all treatment alternatives and SDM target a non-biased access to different treatment options and a treatment decision with regards to patients’ preferences – in dialysis care between HD and PD. But it has not reached routine application in health care yet. For its routine application in health care, comprehensive PD provision might be helpful. SDM is considered to be a valuable tool in clinical medicine and information about different treatment options can pave the way for SDM. Our results show, there is still space for improvement in the provision of information and involving patients to participate in the treatment decision between HD and PD.

## Data Availability

Data are available from the authors upon reasonable request and with permission of DAK-Gesundheit and SBK.

## Abbreviations

CI: confidence interval
DAK: Deutsche Angestellten-Krankenkasse
ESRD: end-stage renal disease
HD: hemodialysis
PD: peritoneal dialysis
SBK: Siemens Betriebskrankenkasse
SDM: Shared decision making
SHI: statutory health insurance
